# Iridescent colouration of male Anna’s hummingbird (*Calypte anna*) caused by multilayered barbules

**DOI:** 10.1007/s00359-018-1295-8

**Published:** 2018-10-08

**Authors:** Marco A. Giraldo, Juan L. Parra, Doekele G. Stavenga

**Affiliations:** 1 Biophysics Group, Institute of Physics, University of Antioquia, Calle 70 #52-21, AA 1226, Medellín, Colombia; 2 Group of Ecology and Evolution of Vertebrates, Institute of Biology, University of Antioquia, Calle 70 #52-21, AA 1226, Medellín, Colombia; 3 Computational Physics, Zernike Institute for Advanced Materials, University of Groningen, Nijenborgh 4, 9747 AG Groningen, The Netherlands

**Keywords:** Feather iridescence, Courtship, Spectrophotometry, Scatterometry, Optical modelling

## Abstract

The male Anna’s hummingbird features a brightly reddish-pink reflecting gorget, due to large stacks of melanosomes in the feather barbules, arranged in layers separated by keratin. Direct observations together with detailed scatterometry demonstrated that the barbules reflect incident light in an approximately specular manner. The structural colouration is iridescent, i.e. varies with a changing angle of light incidence. Spectrophotometrical measurements of the barbule reflectance and absorbance can be well interpreted with calculated spectra obtained with a transfer matrix method for optical multilayers, using anatomical data and measured refractive index spectra. The organization of the reflectors as a Venetian blind presumably functions to create a high spectral contrast of the male’s plumage during courtship.

## Introduction

Among the many colourful birds, hummingbirds stand out because of their extremely shiny feathers (Greenewalt 1960; Stoddard and Prum 2011; Cuthill et al. 2017). Especially male hummingbirds feature brilliant plumages, which they display during courtship toward females. The male’s colourful plumage, as well as his courtship behaviour, is presumably a product of aesthetic evolution by female choice (Darwin 1871; Prum 2012). The iridescent ornamental feathers are sensitive to diet quality and may serve as honest signals of nutrition to mates or rivals (Doucet and Meadows 2009; Meadows et al. 2012).

The barbules of the brightly reflecting feathers of hummingbirds contain stacks of melanosomes, melanin-containing organelles with large air spaces (Greenewalt et al. 1960a). The barbule material thus has a strongly varying refractive index, resembling that of a multilayered interference reflector (Durrer 1977). Optical modelling on the Fiery topaz (*Topaza pyra*) and Anna’s hummingbird (*Calypte anna*) showed that measured reflectance spectra could be well interpreted as to be created by an optical, dielectric multilayer (Greenewalt et al. 1960a, b). At that time, however, the refractive indices of the main material components of the barbules, keratin and melanin, were ill-known. The refractive index values were assumed to be real and wavelength-independent, not in accordance with the complex refractive index of the strongly absorbing melanin.

Recent measurements on various bird feathers have yielded the data necessary for a detailed analysis of bird structural colouration (Leertouwer et al. 2011; Stavenga et al. 2015). Here we focus on the male Anna’s hummingbird (*C. anna*), which has a strikingly coloured, reddish-pink crown and gorget. The colouration is strongly iridescent, because it is very dependent on the angle of illumination and observation (Doucet and Meadows 2009; Meadows et al. 2011). We performed spectrophotometry, scatterometry, anatomy, and optical modelling to investigate the colouration of the gorget feathers. Our study extends previous treatises, confirming that the melanosome stacks essentially behave as an optical multilayer.

## Materials and methods

### Animals and microphotography

Feathers of the gorget of Anna’s hummingbird (*C. anna*) were obtained from the Museum of Vertebrate Zoology (UC Berkeley; sample size 7 feathers). Intact feathers were photographed with an Olympus stereoscope (SZX16 Stereo Zoom Microscope) and an Olympus SC-30 digital camera. Single barbs, mounted on a rotatable stage, were photographed with a Zeiss Universal Microscope (Zeiss, Oberkochen, Germany) using Zeiss Epiplan objectives (16×/0.35 or 40×/0.85) and a Kappa DX-40 (Kappa Optronics, Gleichen, Germany) digital camera. Images of barbs with barbules in oil immersion were taken using a Zeiss 100× oil (NA 0.9) objective.

### Spectrophotometry

Reflectance spectra of the distal, iridescent part of single feathers were recorded with a bifurcated reflection probe (Avantes, Apeldoorn, the Netherlands) using a deuterium–halogen lamp (Avantes AvaLight-D(H)-S) and an AvaSpec-2048 spectrometer (Avantes). A white reflectance standard (WS-2, Avantes) served as a reference. Reflectance spectra were also measured from single barbules with a home-built microspectrophotometer (MSP), which consists of a Leitz Ortholux epi-illumination microscope connected with a fiber optic to the AvaSpec-2048 spectrometer. The light source was a xenon arc and the microscope objective was an Olympus LUCPlanFL N 20×/0.45. Due to the glass UV-absorption, the MSP spectra were limited to wavelengths > 350 nm. Transmittance spectra were measured with the MSP from barbules immersed in oil to reduce light scattering, and were subsequently converted into absorbance spectra.

### Imaging scatterometry

To investigate the spatial far-field reflection properties, we performed imaging scatterometry on small pieces of barbs with about ten barbules on each side (Stavenga et al. 2009). The pieces, attached to the tip of a glass micropipette, were positioned at the first focal point of the ellipsoidal mirror of the imaging scatterometer. Scatterograms were obtained by focusing a white light beam with a narrow aperture (< 5°) onto a circular spot with a diameter of ~ 30 µm to illuminate isolated barbules. The spatial distribution of the far-field scattered light was recorded with an Olympus DP70 digital camera (Olympus, Tokyo, Japan).

### Electron microscopy

A Philips XL-30 scanning electron microscope (SEM) was used to investigate the structure of the barbules and their arrangement on the barbs. To reveal the morphology of the barbules, they were transversally cut with a razor blade, placed on a carbon stub holder, and then sputtered with gold (5–10 nm thickness). For transmission electron microscopy we cut feather barbs from the distal portion of a gorget feather and incubated them in 0.25 M sodium hydroxide and 0.1% Tween 20 for 30 min on a bench-top shaker. These barbs were then transferred to a 2:3 (v/v) solution of formic acid and ethanol for 2.5 h. Feather barbs were dehydrated by incubating in 100% ethanol twice (20 min) and 100% propylene oxide once (20 min) and infiltrated with Epon 812 in successive concentrations of 15, 50, 70 (48 h each) and 100% (24 h). We placed the barb sections into molds with the most distal tip of the barb at the top of the mold, and then the blocks were cured in an oven at 20 °C for 24 h. Finally, we sliced the molds using a diamond knife on an RMC MT-X ultramicrotome (Boeckeler Instruments, Tucson, AZ) to obtain transversal sections relative to the barb. Sections were placed on 200 mesh copper grids (EMS, Fort Washington, PA) with formvar support, post-stained in osmium and lead citrate, and viewed on a Philips EM301 (Veeco FEI Inc, Hillsboro, OR) between 6000 and 60.000× magnification.

### Optical modelling

We calculated the reflectance and absorbance spectra of model barbules with a transfer matrix program based on classical optical multilayer theory, written in Matlab (Stavenga 2014). The model barbules were stacks of melanosomes, embedded in a keratin matrix (Fig. 6c). Based on the anatomy, we used the following parameter values: thickness of the melanosome membrane *a* = 30 nm; thickness internal air layer of top melanosomes *b*_1_ = 20–50 nm; thickness internal air layer of other melanosomes *b*_2_ = 100–125 nm, thickness keratin cortex *c* = 5 nm; thickness keratin layer in between melanosomes *d* = 50 nm; number of melanosome layers was generally taken to be 12, except for Fig. 8c where the number was varied. In the melanosome layers, each melanosome plus surrounding keratin was assumed to occupy a square with area (1000 nm)^2^, with an outside keratin fraction *f*_k_ = 0.05, or, the melanosome area was a square with side *w* = 975 nm. Thin walls inside the melanosomes, perpendicular to the membrane surface, with thickness *e* = 10 nm, divided the internal space into air chambers. Taking *N* = 10 internal walls in a cross-section, or a mesh of (*N* + 1)^2^ = 121 square air holes, the resulting fractions of melanin and air in the internal layer were *f*_m_ = 0.40 and *f*_a_ = 0.55. The real parts of the wavelength-dependent refractive indices of the two components, *n*_k_ and *n*_m_, were calculated with the Cauchy formula *n* = *A* + *Bλ*^−2^ (*λ* is the light wavelength), using for keratin *A*_k_ = 1.532 and *B*_k_ = 5890 nm^−2^ and for melanin *A*_m_ = 1.648 and *B*_m_ = 23,700 nm^−2^; the imaginary component of the refractive index of keratin was assumed to be negligible in the wavelength of interest, but that of melanin was taken to be *k*_m_ = *a*_m_ exp(− *λ*/*b*_m_), with *a*_m_ = 0.56 and *b*_m_ = 270 nm (Leertouwer et al. 2011; Stavenga et al. 2015). The barbule was sliced into 10 nm thick layers and together with the volume fractions of keratin (*f*_k_), melanin (*f*_m_) and air (*f*_a_), with *f*_k_ + *f*_m_ + *f*_a_ = 1, the effective refractive index of each slice, and thus the barbule’s refractive index profile, followed from

1 $${n_{{\text{eff}}}}={f_{\text{k}}}{n_{\text{k}}}+{f_{\text{m}}}(~{n_{\text{m}}} - i{k_{\text{m}}})+{f_{\text{a}}}{n_{\text{a}}}.$$

## Results

### Spectral and spatial characteristics

**Fig. 1:**
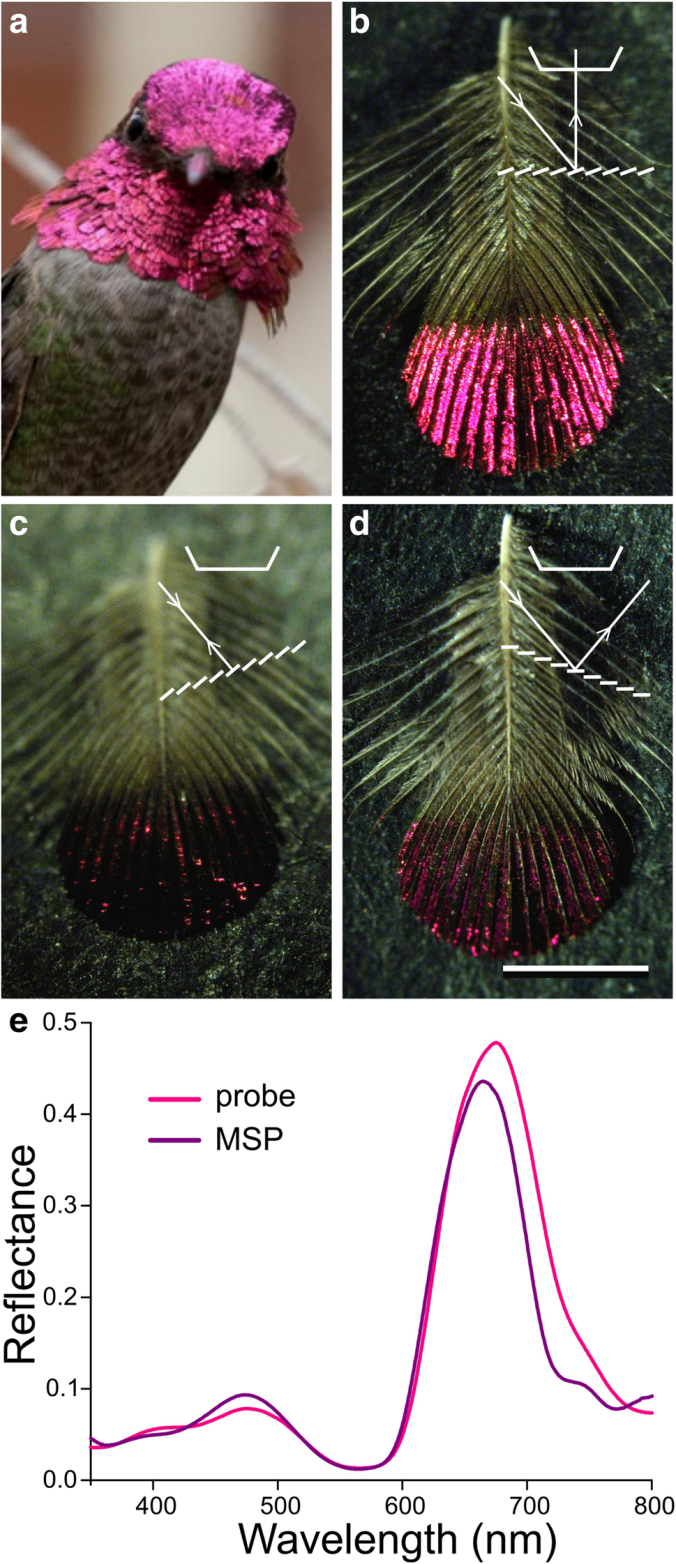
The bright reddish-pink colouration of Anna’s hummingbird. **a** The gorget and crown feathers feature the same colour. **b** An isolated gorget feather showing that only the distal part is coloured. **c** The same feather rotated over − 20° around an axis perpendicular to the feather’s rachis. **d** The feather rotated over + 20°; scale bar (**b**–**d**): 2 mm. Insets: diagrams of obliquely incident light reflected by the feather barbules into the aperture of the microscope objective (**b**) and outside the aperture (**c, d**). **e** Reflectance spectra of the distal area of an isolated feather measured with a bifurcated probe and with a microspectrophotometer (MSP)

The gorget and crown of *C. anna* are covered by bright, reddish-pink feathers (Fig. 1a). The colour is restricted to the distal feather part (Fig. 1b). An isolated gorget feather, positioned with the feather plane about perpendicular to the axis of the observation microscope, showed a maximal reflection when illuminated by a fiber light source from an angle of 40°, meaning that the reflecting elements are rotated by an angle of about 20° with respect to the feather plane (Fig. 1b). The bright reflection almost fully vanished upon rotation of the feather around an axis perpendicular to the rachis over − 20° (Fig. 1c) or + 20° (Fig. 1d), thus demonstrating that the feather reflection is about specular, in full agreement with angle-dependent reflectance spectra measured from iridescent feathers of Anna’s hummingbird by Meadows et al. (2011).

The reddish-pink colour indicates that the feather reflects red light as well as some blue. Measurement of the reflectance spectrum with a bifurcated probe indeed showed a main reflectance band in the red wavelength range, peaking at ~ 670 nm (halfwidth ~ 100 nm), together with a minor band in the blue, peaking at ~ 480 nm (Fig. 1e). Measurements with a microspectrophotometer from small (10 µm)^2^ areas yielded very similar spectra, although the bandwidth was slightly narrower (Fig. 1e), demonstrating that the probe spectra are averages of somewhat variable local spectra.

**Fig. 2:**
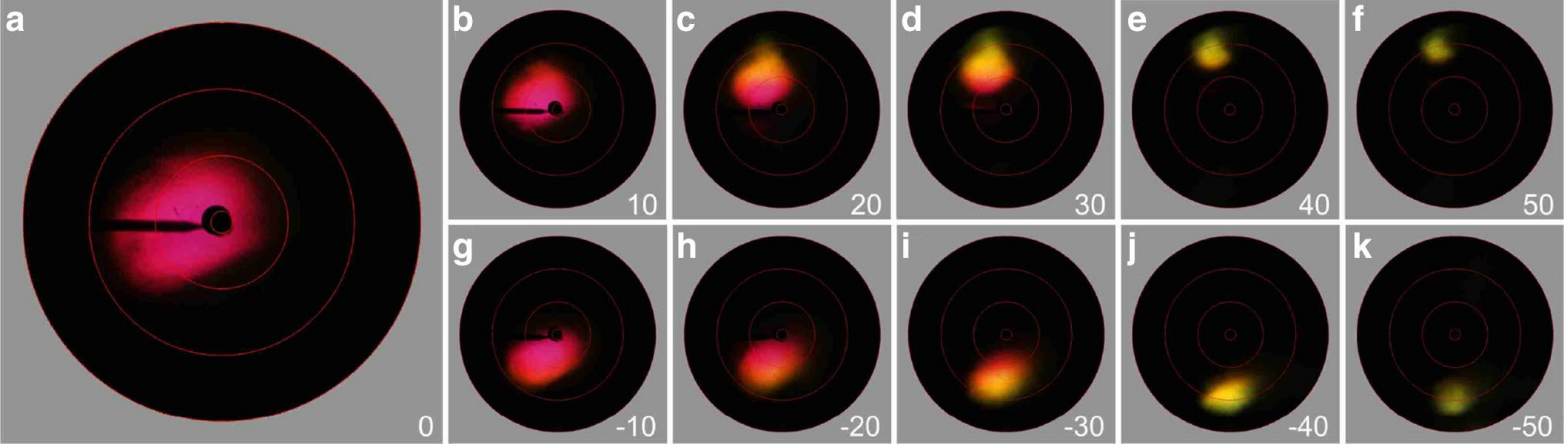
Scatterometry of a small area of the distal part of the feather of Fig. 1b–d. **a** Scatterogram obtained from a slightly tilted (~ 20°) feather applying a narrow-aperture, white-light beam causing reflection into about the axial direction (~ 0°) of the scatterometer. **b**–**f** Scatterograms resulting from illuminations when the angle of incidence was changed in steps of ~ 10° upwards (0° − 50°). **g**–**k** Scatterograms resulting from illuminations when the angle of incidence was changed in steps of ~ 10° downwards (− 10° to − 50°). The red circles indicate scattering angles of 5°, 30°, 60°, and 90°; the black bar at 9 o’clock is the shadow of the glass pipette holding the feather piece

Scatterometry of a small distal piece of the feather confirms the directionality of the reflection. A narrow-aperture, white light beam illuminating the feather piece about normally caused a spatially restricted, reddish-pink scatterogram (Fig. 2a). When the illumination’s angle of incidence was increased, the specularity of the feather was maintained, but the colour changed to shorter wavelengths (Fig. 2b–k). Such a short-wavelength shift is characteristic for an optical multilayer, suggesting the presence of a multilayer-like structure in the hummingbird feathers.

### Structural colouration and pigmentation

**Fig. 3:**
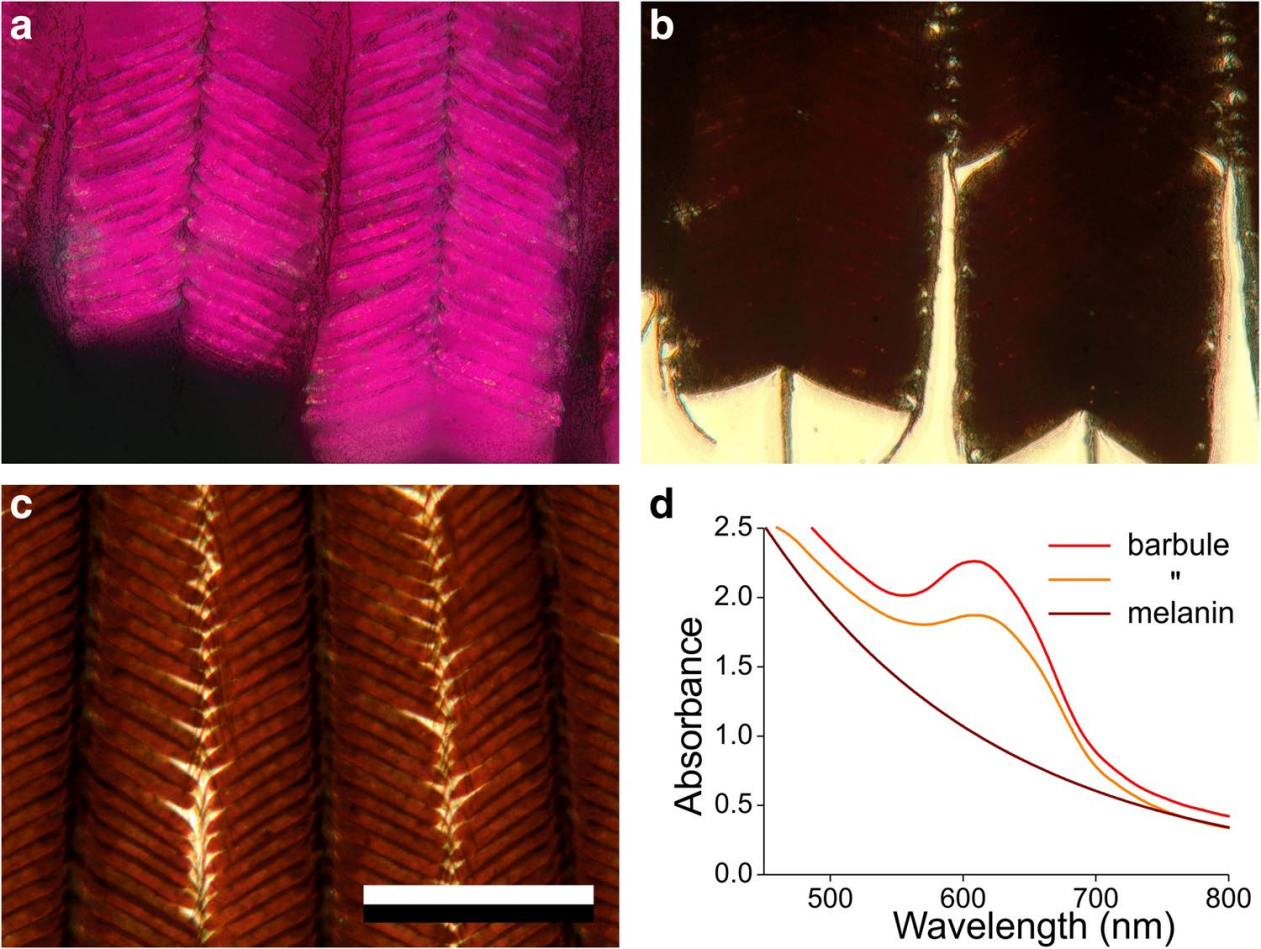
Micrographs of a feather piece in reflected and in transmitted light, and barbule absorbance. **a** Epi-illumination micrograph of a few barbs with barbules, showing highly red-pinkish reflecting barbules. **b** Transmitted-light micrograph showing very dark-brown barbules (in air). **c** Transmitted-light micrograph of barbules in oil immersion; scale bar (**a**–**c**): 200 µm. **d** Absorbance spectra of two barbule locations and the absorbance spectrum of pure melanin

Inspection of the feathers with an epi-illumination microscope demonstrated that the coloured reflections emerge from the barbules (Fig. 3a). Observation with transmitted light yielded a very different picture, indicating a highly absorbing material in the barbules (Fig. 3b). To show that this was not an artifact due to severe scattering, we immersed a feather piece in oil, yielding strongly brown-coloured barbules, which unmistakably indicated the presence of a considerable amount of melanin (Fig. 3c). Transmittance microspectrophotometry of the immersed barbules demonstrated a very high absorbance at short wavelengths, decreasing with increasing wavelengths, except for a distinct absorbance band peaking at ~ 600 nm (Fig. 3d). The latter is most likely related to the distinct reflectance peak in the red wavelength range of Fig. 1e, because an enhanced reflectance causes a reduced transmittance, or, an enhanced absorbance.

To ascertain that the steep increase in absorbance at the shorter wavelengths was indeed due to melanin, we considered that the absorbance of an object, thickness *d*, containing eumelanin with concentration *C*, equals *D*(*λ*) = *ε*_0_ *Cd*exp(− *λ*/*λ*_m_), with ${\varepsilon _0}$ = 2.45 µm^−1^ M^−1^, and ${\lambda _{\text{m}}}$ = 175 nm (Stavenga et al. 2012). We approximated the measured absorbance spectra of Fig. 3d with the spectrum $D\left( \lambda \right)=33{\text{exp}}\left( { - \lambda /175} \right)$. Assuming a concentration *C* = 7.9 M of melanin, similar as previously derived for the densely pigmented wings of the male damselfly *Calopteryx japonica* (Stavenga et al. 2012), we obtain an effective thickness of 1.7 µm for the pigment in the barbules of Anna’s hummingbird gorget feathers. When the effective melanin thickness per melanosome is 97 nm (see “Materials and methods”), this would suggest that 17.5 layers of melanosomes exist. This value is in reasonable correspondence with the anatomy showing stacks of 12–15 orderly arranged melanosome layers together with a few additional, randomly organized melanosomes (see below).

**Fig. 4:**
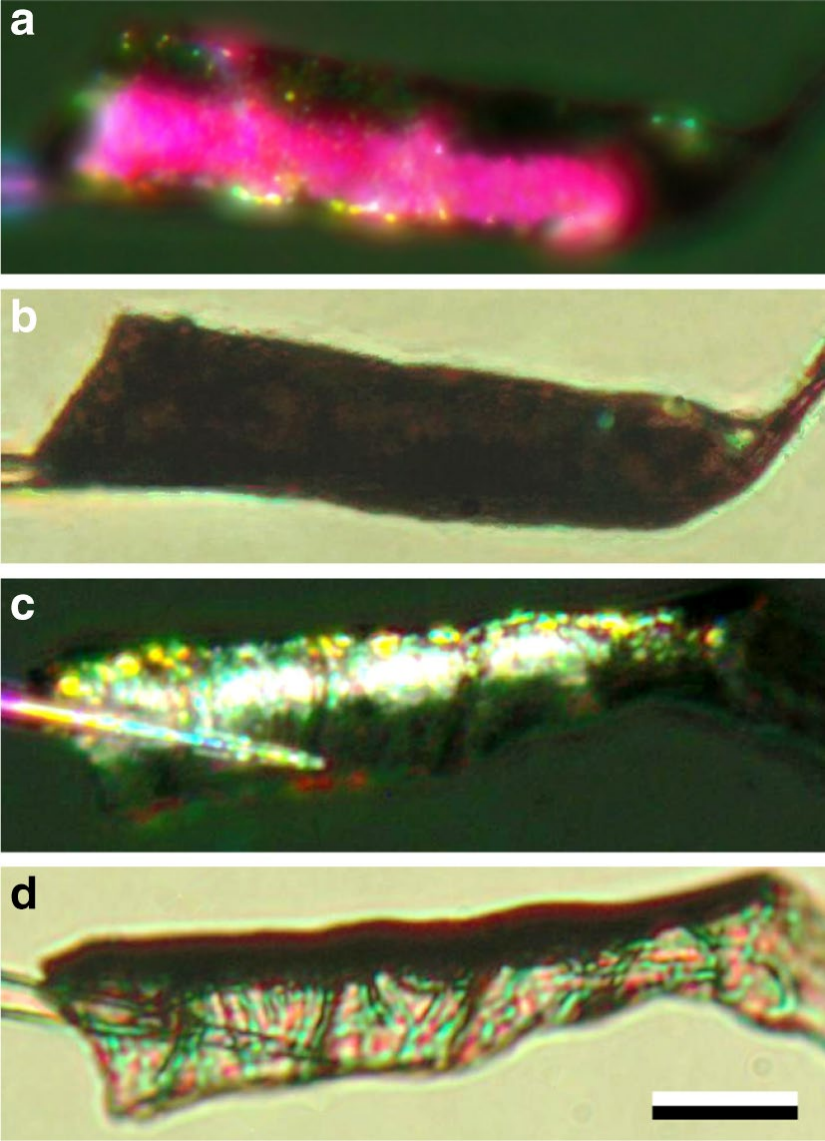
An isolated barbule glued to a glass pipette. **a** Epi-illumination creating a bright reddish-pink reflection from the upper lamina. **b** The same situation but in transmitted light. **c** Epi-illumination of the barbule rotated by 80°. **d** The latter situation, but in transmitted light; scale bar (**a**–**d**): 20 µm

We further investigated the structural properties of the barbules by isolating a single barbule and gluing it to the tip of a glass micropipette. The barbule appeared to be a long, longitudinally folded blade with a hook at the end. Only when properly oriented, epi-illumination of the barbule caused the bright reddish-pink colour (Fig. 4a, cf. Fig. 3a); in transmitted light the barbule was very dark-brown (Fig. 4b, cf. Fig. 3b, c). Upon rotation of the barbule over 80°, to expose the side lamina, epi-illumination created a broad-band whitish reflection (Fig. 4c), but in transmitted light the side lamina appeared to be rather transparent, with erratic interference colours, presumably due to thin film effects (Fig. 4d).

### Anatomy of the barbules of the male Anna’s hummingbird

**Fig. 5:**
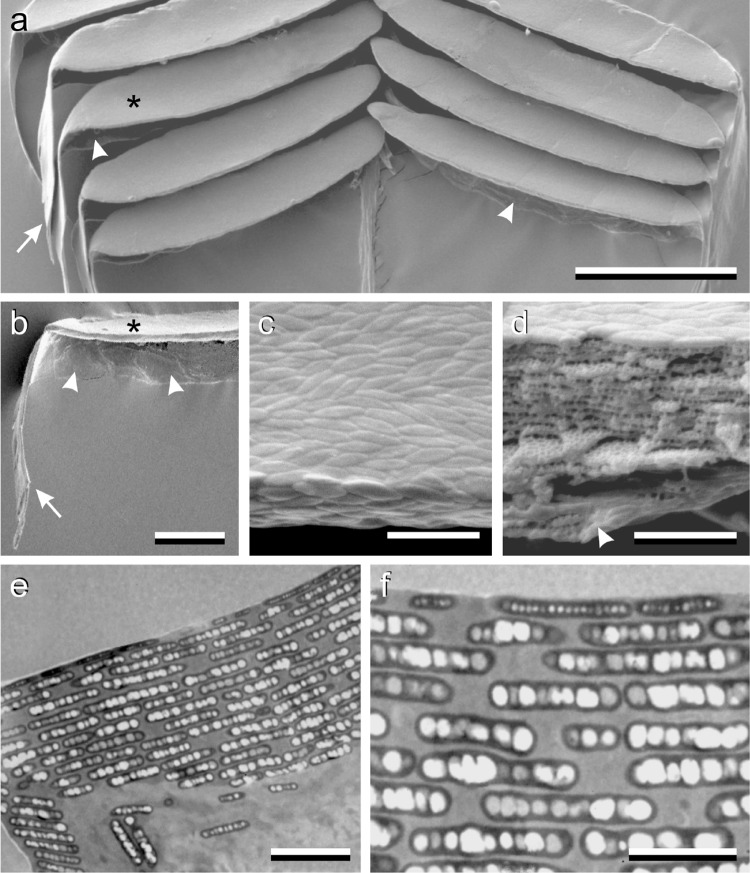
Electron microscopy of hummingbird barbules. **a** Scanning electron micrograph of barbules attached to both sides of a barb. Each barbule is a folded plane with an exposed lamina (asterisk) and a pronounced (hidden) side wall (arrowheads), and at the end a long hook (arrow). **b** A single barbule showing the lamina (asterisk), side wall (arrowheads) and hook (arrow). **c** Both the top and lateral surfaces are marked by a mosaic consisting of spindle-shaped structures. **d** A cut barbule revealing layers with air holes in the main lamina. **e** Transmission electron micrograph of the transition edge area of the upper lamina and the side wall, showing numerous layers of melanosomes. **f** The upper layers of melanosomes, within the uppermost layer much smaller melanosomes than those in the other layers; scale bars: **a** 50 µm, **b** 20 µm, **c**–**e** 2 µm, **f** 1 µm

The detailed shape and organization of the barbules became clear by performing electron microscopy. Scanning electron microscopy (SEM) demonstrated that the upper laminae of the barbules are angled with respect to the feather’s plane, but parallel to each other as the lamellae in a Venetian blind (Fig. 5a). Both the exposed and side laminae have a length of ~ 100 µm and a width of ~ 20 µm (Fig. 5a,b, asterisks and arrowheads). The side sheet is prolongated into a more or less perpendicularly oriented hook with length ~ 60 µm (Fig. 5a,b, arrows). Close inspection showed that the upper lamina surface and its rim are marked by an irregular mosaic of spindle-shaped structures, length 1.1 ± 0.2 µm and width 0.24 ± 0.04 µm (Fig. 5c). Cutting the barbules showed that the upper lamina consists of 12–15 layers, with periodicity ~ 210 nm, containing numerous holes (Fig. 5d).

The layers are especially clearly seen in transmission electron microscope (TEM) sections, revealing that the layers are stacks of coplanar arranged melanosomes (Fig. 5e, f). The TEM micrographs show a very thin cortex. The upper layer of melanosomes is pressed against the cortex, explaining the corrugated barbule surface (Fig. 5c). Quite noticeably, the melanosomes in the upper layer are much thinner than the ones in the layers below (Fig. 5e, f), and furthermore SEM micrographs also indicated that their surface area is distinctly smaller than that of the lower ones (e.g. Fig. 5d). The period of the melanosome stack shown in the TEM images is 300–330 nm (Fig. 5e, f), much higher than the value ~ 210 nm derived by SEM (Fig. 5d). Because optical modeling (see below) yielded period values of 210–230 nm, in agreement with the SEM data, we conclude that the TEM sections were skew to the barbule surface.

**Fig. 6:**
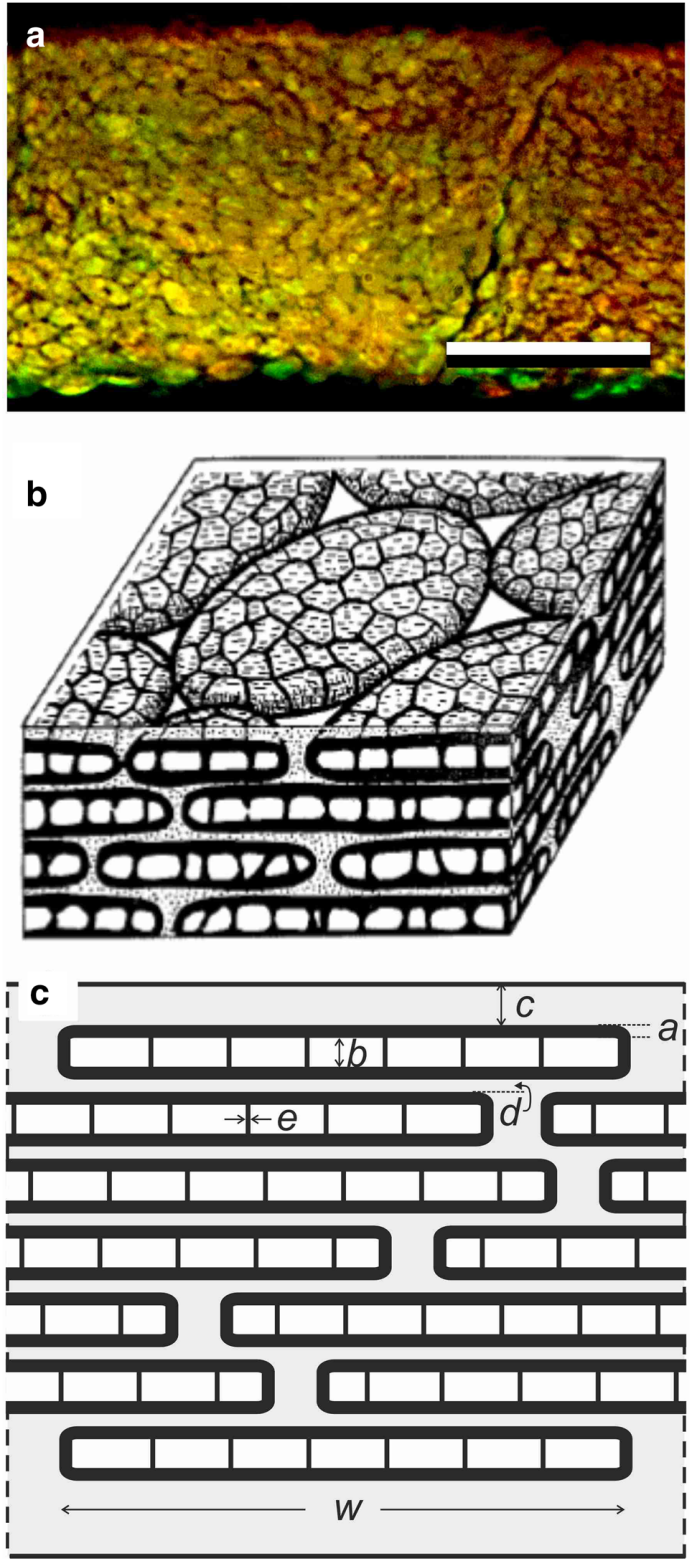
Melanosome stacking in hummingbird barbules. **a** Epi-illumination micrograph of a barbule immersed in oil showing 1.5 cells; scale bar: 10 µm. **b** Drawing based on TEM anatomy (Fig. 42 from Durrer 1977). **c** Diagram showing the parameters used in the modeling (see “Materials and methods”)

To study the arrangement of the melanosomes more directly, we performed epi-illumination of small feather pieces immersed in oil (Fig. 6a). The reflection images showed a rather disordered pattern, similar as the SEM images (Fig. 5c). The bright areas evidently represented reflecting melanosomes, but in between numerous dark spots existed, indicating interstitial gaps. The pattern of the light micrograph is well compatible with Durrer’s diagram (Fig. 6b) of the inner workings of hummingbird barbules (Durrer 1977). We have abstracted this spatial diagram into the model barbule of Fig. 6c, which we used in the computational modeling presented below.

### Modelling the barbule’s structural colouration

**Fig. 7:**
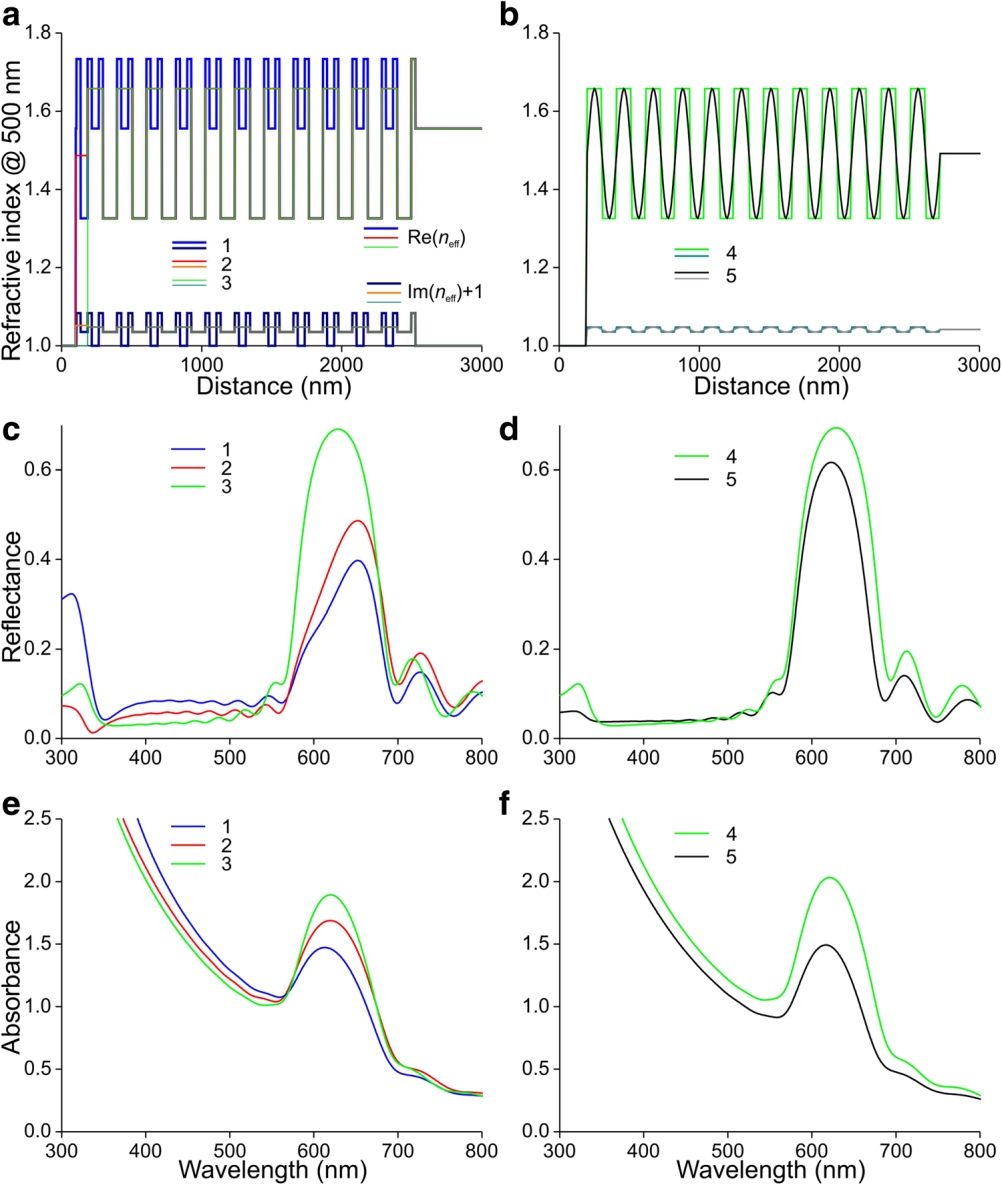
Modeling the optical properties of a barbule. **a, b** Real and imaginary parts of the effective refractive index (RI) profile at 500 nm, Re(*n*_eff_) and Im(*n*_eff_). **c, d** Reflectance spectra resulting for the cases 1–5 for normally incident light. **e, f** Absorbance spectra resulting for cases 1–5. Case 1: A stack of 12 melanosomes. The RI is high at the melanosome membrane level, lower at the level of the keratin intermediate of the melanosomes, and much lower at the level of the air compartments of the melanosomes. Case 2: The RIs of two adjacent membranes and the intermediate keratin are averaged, and also the average is taken of the RIs in the frontal part, existing of the cortex layer, the distal membrane as well as the layer of the inner part of the upper melanosome. Case 3: The frontal part is neglected, resulting in 11 periods of a square wave plus a final RI pulse of a membrane layer. Case 4: Similar to case 3, except for the replacement of the last membrane layer by another square wave period. Case 5: A sinusoidally oscillating RI with extreme values identical to those of case 4

We calculated the reflectance and absorbance spectra for normally incident light, considering five special cases of barbules (Fig. 7). In case 1, we studied an idealized barbule with 12 layers of melanosomes. A thin (5 nm) cortex layer of pure keratin covered the layers containing melanosomes, with membrane thickness 30 nm and in between 100 nm thick air compartments. The melanosome layers were identical, except for the upper melanosome layer, where the melanosomes had 50 nm thick air compartments. A keratin layer of 50 nm separated the melanosome layers, so that the stack period was 210 nm (Fig. 6c). We calculated the effective refractive index along a coordinate normal to the barbule surface, using previously determined refractive index spectra, as described in “Materials and methods”. The refractive index of the keratin cortex and the sheets in between the melanosome layers was real, but because the melanosome membranes as well as their internal walls contained melanin, the effective refractive index of the melanosome layer was complex (Fig. 7a, b). We implemented the above parameters in a transfer matrix program and calculated the reflectance spectrum as a function of wavelength, resulting in a spectrum with a distinct peak in the red and a broadband in the blue wavelength range (Fig. 7c). The simultaneously calculated absorbance spectrum also peaked in the red, although hypsochromic shifted (towards shorter wavelengths), and the absorbance sharply increased with decreasing wavelength, as expected (Fig. 7e, f, cf. Fig. 3d).

The anatomy of Fig. 5 clearly shows that neither the cortex surface nor the melanosome layers are perfectly flat, and therefore, we considered case 2, where first the refractive index values of the three top layers (the cortex, the upper membrane of the first melanosome layer and its thin air layer) were averaged and secondly the refractive index values of the two closely opposed membranes of adjacent melanosome layers together with the intermediate keratin layer were averaged (Fig. 7a). This resulted in reflectance and absorbance spectra with increased red peaks (Fig. 7c, e). Apparently the refractive index jump at the barbule surface plays a crucial role, because when we neglected the top layers (case 3, Fig. 7a), the red peaks of both the reflectance and absorbance increased substantially (Fig. 7c, e).

The refractive index profile of case 3 resembles a square wave, except for the last bit of membrane (Fig. 7a). We, therefore, considered case 4, which has 12 square waves with extreme values identical to those of the square wave of case 3. Image analysis of the electron micrographs indicated that a sinusoidally varying refractive index may be a more realistic representation of the barbule’s refractive index profile. For instance, the barbule surface is slightly corrugated and the upper melanosome layer is not homogeneously filled with melanosomes (Fig. 5d–f), and hence the effective refractive index will not change abruptly in the barbule surface layer. The melanosomes are also not arranged in perfectly flat layers, which will cause smoothing of the refractive index profile. We, therefore, compared case 4 with case 5, a sine wave (Fig. 7b). The reflectance and absorbance spectra calculated for both cases appeared to be rather similar, except for a slight difference in magnitude (Fig. 7d, f).

**Fig. 8:**
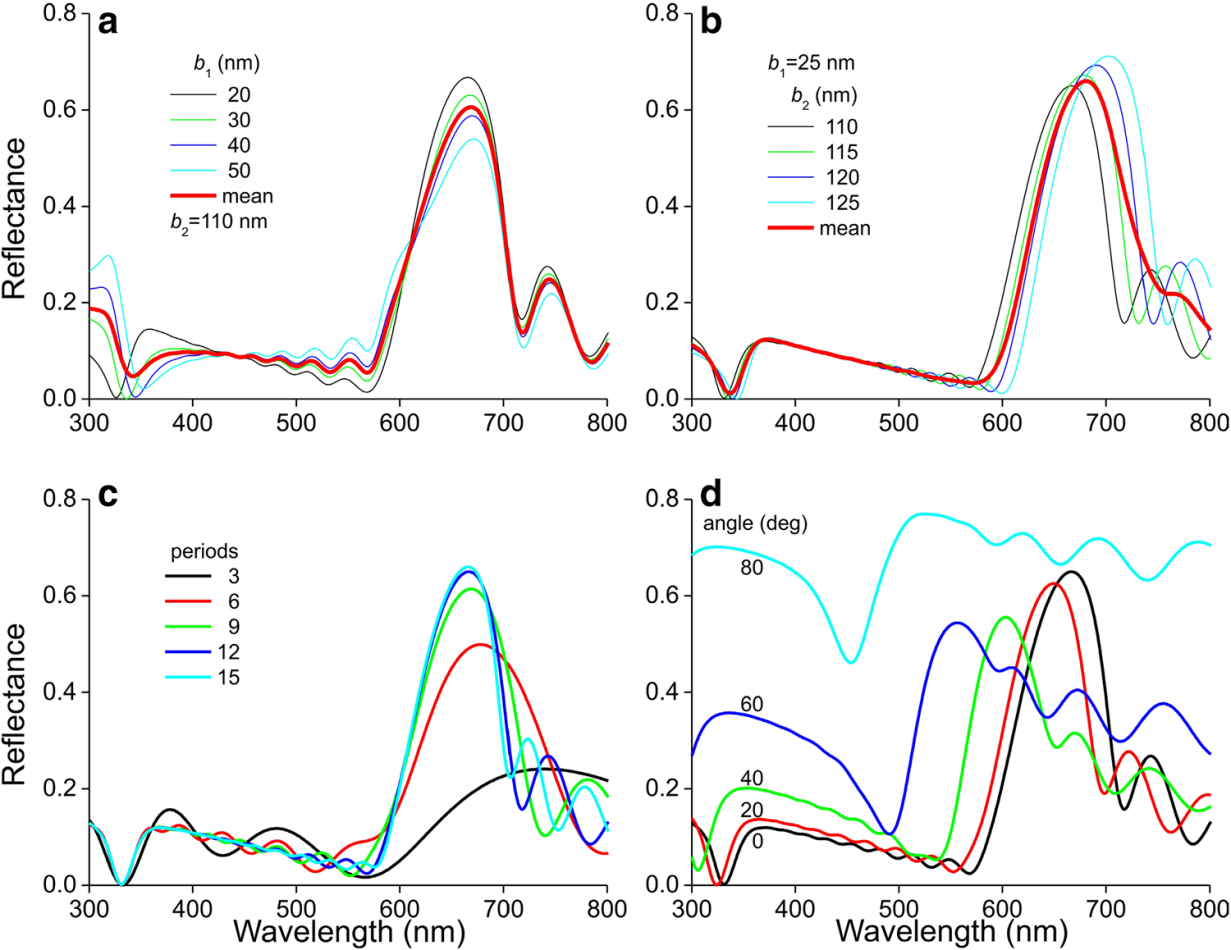
Reflectance spectra of a barbule with various parameters. **a** Dependence of the reflectance on the thickness of the top melanosome layer; normally incident light, 12 layers (*b*_1_: thickness of air layer of first melanosome layer; *b*_2_: thickness of air layer of the second to last melanosome layer). **b** Dependence of the reflectance on the thickness of the melanosome layers below the top layer. **c** Dependence of the reflectance on the number of periods (layers). **d** Dependence of the reflectance on the angle of light incidence of unpolarized light; 12 layers (**c, d**: *b*_1_ = 25 nm, *b*_2_ = 110 nm)

The prominent reflectance band in the modeled spectra in the red wavelength range resembles the main band in the measured reflectance spectra (Fig. 1e), but the sideband in the blue wavelength range of the latter spectra was not reproduced. As the top melanosome layer strongly differed from the other layers, we investigated the possibility that the shape of the reflectance spectra is subtly sensitive to the properties of the first melanosome layer. We varied the thickness of the first air layer *b*_1_ from the initially chosen value of 50 nm. A distinct reflectance valley emerged at decreasing *b*_1_ values (Fig. 8a). Assuming thus *b*_1_ = 25 nm, this valley appeared to be insensitive to the thickness of the air layer in the inner melanosomes, *b*_2_, when varied from 110 to 125 nm (Fig. 8b).

We furthermore investigated the dependence of the reflectance on the number of periods (i.e. layers), using the values *b*_1_ = 25 nm and *b*_2_ = 110 nm. The reflectance bandwidth is large with only a few melanosome layers but rapidly narrows with an increase in layer number (Fig. 8c). With more than 10 layers the peak reflectance stabilizes and the bandwidth of ~ 100 nm corresponds well with the experimental data. This is classical behaviour for biological multilayer reflectors (Land 1972; Wilts et al. 2009; Stavenga et al. 2017). We finally calculated the dependence of the reflectance spectra on the angle of light incidence for the case with 12 periods. Averaging the spectra for TE- and TM-polarized light yielded the reflectance spectra for unpolarized light. With an increasing angle of light incidence, the red peak strongly shifted towards shorter wavelengths (Fig. 8d).

## Discussion

We have modeled the reflectance of the feather barbules of the male Anna’s hummingbird’s gorget using a transfer matrix method for optical multilayers. We assumed dimensions suggested by anatomy, and we estimated the effective refractive indices of the layers via a simple, weighted average of the refractive indices of the material components. The applied approach allows considerable insight into the essential optical properties of the barbules of hummingbird feathers. For instance, the strong hypsochromic shift occurring upon an increase of the angle of light incidence following from the model calculations (Fig. 8d) is fully in agreement with measured spectra (Meadows et al. 2011) as well as with the colour change from red via yellow to greenish seen in the scatterograms when changing the angle of illumination from normal to 50° (Fig. 2). Furthermore, the calculations show that a stack of more than 10 melanosome layers of the hummingbird barbules creates a high peak reflectance, close to saturation (Fig. 8c). Adding more layers only very slightly increases the peak reflectance and narrows the bandwidth. This will only make sense when extreme brilliance and spectral contrast is very important for signaling, which is apparently the case, given the 12–15 layers seen in the electron micrographs (Fig. 5d-f).

The effective refractive indices used in the modelling were estimated via a simple volume average of the components. The large differences in the refractive index values between melanin, keratin and air may yield slightly deviant effective refractive indices, but the modeled reflectance spectra will be appropriate approximations, certainly compared to the modeled spectra of Greenewalt et al. (1960a, b), who in their pioneering studies on the structural colouration of hummingbird feathers assumed a wavelength independent, real refractive index of melanin (*n*_m_ = 2.0), which we now know is not close to realistic values (Stavenga et al. 2015).

Quite critical are the dimensional values used in the calculations. For instance, a sinusoidal refractive index profile with period 210, 220 or 230 nm produces reflectance spectra with peak wavelengths 623, 652, and 680 nm, respectively. The experimentally obtained reflectance peak wavelength of the gorget feathers was ~ 670 nm, which indicates that the period 210 nm chosen on the basis of anatomical melanosome values, represents a slight underestimate. Generally, hummingbirds display structural colours varying throughout the whole visible wavelength range, from violet-blue to scarlet-red. This can be straightforwardly achieved by adjusting the periodicity of the barbules’ melanosome stacks (Giraldo et al. in prep.).

In our optical modelling, we have not taken into account the space below the melanosome stack. Part of the incident light that has passed the melanosomes will reach the lower surface of the barbule speculum, where it may be partly reflected. In principle that reflected light could contribute to the interference process, but anatomy shows that the lower surface of the barbule is far from flat, so that the reflected light will not substantially contribute to interference, as well as will be spread into a large spatial angle. The reflected light flux can, therefore, be neglected, especially in the shorter wavelength range where melanin absorption is high. In the very long-wavelength range, where melanin absorption is minor, scattering by the randomly arranged melanosomes may contribute a slight background reflectance.

Similar as Greenewalt et al. (1960a), we found that barbules in immersion oil had reflectance spectra with only the main red peak, but no blue secondary band. The modeling of Fig. 8 shows that the top layer with very thin melanosomes plays a crucial role in creating the sideband. A valley in the reflectance spectra emerges when the air layer is assumed to be very minor. However, the calculated blue sideband extends well into the ultraviolet, not completely corresponding with the measured spectra. We hence have not yet reached a fully satisfactory modeled reflectance in the blue wavelength range. We nevertheless believe that the main features of the *C. anna* hummingbird feathers can now be understood in considerable detail.

The structural coloured feathers of hummingbirds are highly specialized. As noted by Durrer (1977), the barbules of bird feathers are usually oriented perpendicular to the feather plane, probably to optimise hydrophobicity (Eliason and Shawkey 2011). To display a structural-coloured barbule, it must be rotated so that it becomes more or less coplanar with the feather surface. Durrer (1977) provides many examples of structural-coloured bird barbules that are curved, but the male Anna’s hummingbird’s barbules appear to be hybrid. They are folded planes, of which the speculum part is about parallel to the feather plane and the other, the velum part, has maintained the about normal, perpendicular orientation (Greenewalt et al. 1960a; Durrer 1977). This allows the barbules of adjacent barbs to preserve their interconnections via the hooks (Durrer 1977). Whereas the velum part is hidden, only the speculum part of the barbule is exposed and thus can have a display function.

The highly specialized features of structurally coloured feathers in birds are assumed to be a product of sexual and natural selection (Darwin 1871; Parra 2010; Prum 2012). One of the most famous examples of the use of structural colouration signals in courtship comes from birds of paradise (family: Paradisaeidae). The barbules of the breast feathers of the male bird of paradise *Parotia lawesii* are boomerang-shaped, and the cortex is a blue-reflecting thin film enveloping a golden-yellow reflecting multilayer of rod-shaped melanosomes (Stavenga et al. 2010; Wilts et al. 2014). During courtship, the male flashes the highly reflecting breast feathers as well as nape feathers towards on-looking females that are seated on an overhanging branch (Laman and Scholes 2012).

Male Anna’s hummingbirds and other close relatives do it quite differently. The male uses two flight displays: dives and shuttles (Stiles 1982). While the former is the most widely known (Clark 2009, 2012), the second seems to be the most critical during courtship (Stiles 1982). During the shuttle display the male often hovers close (< 30 cm) and obliquely above a female with his head pointed towards her, so that the angled barbules maximally reflect incident sky and/or sunlight, spectrally reduced into a narrow wavelength band, toward the female (Stiles 1982, but see also; Simpson and McGraw 2018; https://www.youtube.com/watch?v=scCZ8aoq_sA). It is important to notice then that the reflectance band displayed toward the female is shifted to shorter wavelengths, depending on the angle of light incidence (and reflection); the spectral shift becomes quite noticeable at angles of incidence > 20° (Fig. 8d). Presumably, the reason why the barbules’ speculae are not fully parallel to the feather plane, but rather organized like a closely fitting Venetian blind, is because this way the sky and sun light’s angles of incidence and reflection are reduced, and thus the peak wavelength of the reflected light will remain above 600 nm.

The reflected light spectrum thus about coincides with the spectral sensitivity of the visual system’s red receptor, because similar to other birds, hummingbird vision is most likely tetrachromatic, based on a set of UVS/VS, SWS, MWS and LWS receptors. The peak wavelength of the LWS receptors of all 21 species studied by Hart and Vorobyev (2005) was > 600 nm. Herrera et al. (2008) derived from ERG measurements a peak wavelength 560 nm for the LWS receptor of the green-backed firecrown hummingbird, *Sephanoides sephaniodes*, but that value is presumably an underestimate. A potential advantage of a structure that generates a reflectance spectrum that peaks at a very long wavelength and has a narrow bandwidth is the high chromatic contrast with the background, which has been shown to be used by birds when searching for food (Schaefer et al. 2006) and also by red flowers against their background (Herrera et al. 2008). Thus, this might be another example of sensory exploitation in which the male captures the female’s attention.
